# A Computer-Aided Analysis Method of SPECT Brain Images for Quantitative Treatment Monitoring: Performance Evaluations and Clinical Applications

**DOI:** 10.1155/2017/1962181

**Published:** 2017-01-31

**Authors:** Xiujuan Zheng, Wentao Wei, Qiu Huang, Shaoli Song, Jieqing Wan, Gang Huang

**Affiliations:** ^1^Department of Automation, The School of Electrical Engineering Information, Sichuan University, Chengdu, China; ^2^Department of Nuclear Medicine, Renji Hospital, School of Medicine, Shanghai Jiaotong University, Shanghai, China; ^3^Med-X Institute, Shanghai Jiaotong University, Shanghai, China; ^4^Department of Neurosurgery, Renji Hospital, School of Medicine, Shanghai Jiaotong University, Shanghai, China

## Abstract

The objective and quantitative analysis of longitudinal single photon emission computed tomography (SPECT) images are significant for the treatment monitoring of brain disorders. Therefore, a computer aided analysis (CAA) method is introduced to extract a change-rate map (CRM) as a parametric image for quantifying the changes of regional cerebral blood flow (rCBF) in longitudinal SPECT brain images. The performances of the CAA-CRM approach in treatment monitoring are evaluated by the computer simulations and clinical applications. The results of computer simulations show that the derived CRMs have high similarities with their ground truths when the lesion size is larger than system spatial resolution and the change rate is higher than 20%. In clinical applications, the CAA-CRM approach is used to assess the treatment of 50 patients with brain ischemia. The results demonstrate that CAA-CRM approach has a 93.4% accuracy of recovered region's localization. Moreover, the quantitative indexes of recovered regions derived from CRM are all significantly different among the groups and highly correlated with the experienced clinical diagnosis. In conclusion, the proposed CAA-CRM approach provides a convenient solution to generate a parametric image and derive the quantitative indexes from the longitudinal SPECT brain images for treatment monitoring.

## 1. Introduction

Single photon emission computed tomography (SPECT) with ^99m^Tc-ethyl cysteine dimer (^99m^Tc-ECD) has been widely used to evaluate many types of cerebrovascular diseases and brain disorders by measuring regional cerebral blood flow (rCBF) [1–6]. Moreover, longitudinal ^99m^Tc-ECD SPECT brain imaging can be adopted to monitor the changes of rCBF to support the treatment plan [7, 8]. The most common approach for interpreting SPECT brain images is visual inspection in daily clinical practice. Usually the relevant structural images, such as CT or MRI images, are preferred for the visual interpretation together with SPECT images. For treatment monitoring, the baseline and follow-up SPECT brain images should be parallelly interpreted under the same condition to figure out the differences. Due to the lack of the quantitative standards, the accuracy and reliability of visual inspection mainly rely on the experience of the physicians, such that only the qualitative results are presented in the reports. The aforementioned drawbacks of vissual insspection prevent the applications of SPECT imaging in distant diagnosis and multicenter studies. The image processing technology can bring solutions to the visual inspection problems and explore the hidden information in the images.

Beside the region of interest (ROI)/volume of interest (VOI) analysis methods, statistical parametric mapping (SPM) has been used in group-wise comparisons of functional brain images to evaluate the responses to the treatments [9, 10]. On the other hand, subtraction analysis is also a useful technology to extract differences in a series of images for individual treatment monitoring. In ictal-interictal SPECT imaging, the subtraction analysis has been proved to benefit the treatment plan of epilepsy patients [11–14]. Recent studies have reported that the quantitative SPECT analysis would be playing an ever-growing role in treatment plan and response monitoring of several disorders related with the central nervous system [15, 16]. These studies encourage us to generate the parametric imaging and extract quantitative indexes from the SPECT images to support the treatment plan. In this study, we introduce a computer-aided analysis (CAA) method inherited from subtraction analysis to quantify the changes of rCBF in longitudinal SPECT images for individual treatment monitoring. The performance of the proposed method would be objectively and systematically evaluated by the computer simulations and the clinical applications.

## 2. Materials and Methods

### 2.1. Computer-Aided Analysis Method

**Figure 1:**
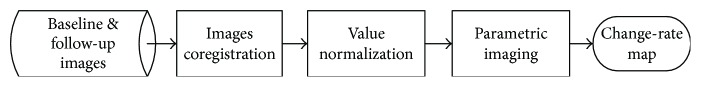
The workflow of the computer-aided analysis method to extract a change-rate map.

When using SPECT imaging in treatment monitoring, the pre- and postscans are usually performed to acquire the baseline and follow-up SPECT images before and after delivering treatments. The obtained baseline and follow-up images from an individual subject are a set of longitudinal SPECT images requiring independent analysis. For interpreting the individual subject's data, a computer-aided analysis (CAA) method is established to process the longitudinal SPECT images via three main steps: coregistration, value normalization, and parametric imaging. The workflow chart is shown in Figure 1.

In the first step, the follow-up SPECT brain images are aligned with the baseline images by the rigid registration algorithm provided by SPM 8.0 software package (http://www.fil.ion.ucl.ac.uk/spm/). If the SPECT brain imaging is performed using SPECT-CT integrated system, the CT and SPECT images can be obtained in the same position and considered as well aligned. In this condition, the baseline and follow-up CT images could be used as the reference and source image, respectively, in the step of coregistration for aligning longitudinal SPECT images.

In the second step, the value normalization is applied on the SPECT images. Before the numerical calculation, the extracerebral voxels are removed by a predefined whole-brain mask. If only the SPECT image is available, then the whole-brain mask can be obtained by segmenting the enhanced SPECT image with Otsu's algorithm. If the corresponding aligned CT image is available, then the whole-brain mask can be more accurately defined by separating the brain tissues from nonbrain tissue in CT images using fuzzy *C*-means clustering algorithm [17]. The whole-brain mask extracted based on the CT images is then applied on the SPECT images to delineate the brain area.

After deriving the whole-brain area in the SPECT images, the value of each cerebral voxel is normalized by the average voxel value of the reference area that is automatically selected by *Z*-map approach [18]. In *Z*-map approach, the *Z* value of the *i*th voxel was calculated as in (1). Two *Z*-maps are, respectively, estimated for baseline and follow-up SPECT brain images. Then, the reference region is the intersection of the *Z* < 1 areas of these two *Z*-maps.

(1) $$\begin{array}{c} Z_{i}=\frac{\left|C_{i}-mean\right|}{SD}, \end{array}$$

where *C*_*i*_ is the value of the *i*th voxel in one SPECT brain image; mean and SD, respectively, denote the average and standard deviation of voxel values of brain area in the SPECT image.

In the third step, the changes in the longitudinal SPECT images are expressed in a parametric image to reflect the disease progress or responses to the treatment. As in subtraction analysis, the difference can be directly obtained by subtracting two aligned normalized images, as

(2) $$\begin{array}{c} D_{i}=C_{i}^{f}-C_{i}^{b}, \end{array}$$

where *C*_*i*_^*f*^ and *C*_*i*_^*b*^ denote the normalized values of the *i*th voxel in the follow-up and baseline SPECT images, respectively.

Next, the change-rate map can be calculated voxel-by-voxel to reflect the extent of the changes between the baseline and follow-up images. The value of the *i*th voxel in the estimated change-rate map is denoted by *R*_*i*_, which can be derived from

(3) $$\begin{array}{c} R_{i}=\frac{D_{i}}{C_{i}^{b}}. \end{array}$$

The change-rate map (CRM) is a parametric image which can be fused with aligned SPECT/CT images for visual inspections. The positive voxel value in CRM demonstrates the recovery of hypoperfusion, while the negative value indicates the recovery of hyperperfusion. For the visualization, Gaussian smoothing filter can be applied in the CRM to reduce the impact of noise. Based on the CRM, the changed regions are automatically obtained by thresholding and clustering. Firstly, the voxels with lower change-rate (<20%) were set to 0 in CRM. Then, the *K*-means clustering algorithm is adopted to recognize the changed regions. The morphological processing is applied to refine and distinguish each region. Considering the SPECT image resolution and partial volume effects, the regions with larger volumes (>120 voxels) are selected as the recovered regions.

For the localization of recovered regions, an atlas of brain lobes, which consists of 12 brain anatomical structures (listed in Table 1), is created from the Talairach Daemon atlas [19, 20] and then translated into MNI (Montreal Neurological Institute) space [21] with dimensions of 91 × 109 × 91 sampled at 2 mm intervals, corresponding to the SPM templates [22]. The SPECT/CT images as well as the obtained CRM are mapped to SPM template by the nonrigid registration algorithm provided by SPM 8.0 software package and then aligned with the atlas of brain lobes. Based on the atlas of brain lobes, the recovered regions could be located in the different brain lobes. The quantitative indexes for the recovered regions, such as the mean and maximum change-rate and the proportion of the recovered regions to the corresponding brain lobes, could be calculated for each detected recovered regions.

In order to facilitate the expression, the proposed approach used to evaluate longitudinal SPECT images through a CRM derived by the CAA method is noted as CAA-CRM approach in the subsequent parts.

### 2.2. Computer Simulations

#### 2.2.1. Simulated Data

In this study, the performance of the CAA-CRM approach in treatment monitoring was objectively and systematically evaluated by the computer simulations. The longitudinal SPECT images are simulated using predesigned digital brain phantoms. The normal digital brain phantom is a 100 × 100 × 82 matrix representation of the hardware Hoffman phantom [23], whose voxel size is 2.13 mm × 2.13 mm × 2.13 mm. In the digital brain phantom, the value of each voxel presents the radioactivity in the corresponding position. Generally, this digital brain phantom is used to simulate the normal brain perfusion images acquired by ^99m^Tc-ECD SPECT imaging. Comparing with other regular geometrical objects, the sphere is more suitable for simulating the ischemic lesions in perfusion images. For convenience, a sphere is created in the normal digital brain phantom located in the right frontal lobe as a lesion analogue. The diameter and radioactivity of the sphere can be changed for several scales to simulate the varied degrees of hypoperfusion for brain ischemia. The diameter of the sphere was designed in three scales: 8 mm, 16 mm, and 24 mm. In addition, the radioactivity in the sphere was set based on the predefined change-rate scaled in 9 different levels uniformly distributed from 10% to 90%. The simulated brain SPECT images are generated with an injected dose of 25 mCi of ^99m^Tc-ECD. The abnormal and normal brain images are, respectively, regarded as the baseline and follow-up images obtained in treatment monitoring for brain ischemia.

The system parameters used in computer simulations are set according to the geometry of the dual-head Philips Precedence 6 SPECT/CT scanner. Each detector head is mounted with a low-energy and high-resolution (LEHR) collimator. The two heads rotate in H-mode to obtain 128 projections in total over 360° around the predesigned phantom. Projection data are acquired for 10 minutes with a total count number of 10^8^ accompanied with measurement noise that is modeled as an additive Poisson noise. The radioactivity distribution in the brain phantom is reconstructed with a maximum a posteriori (MAP) algorithm with total variation regularization.

#### 2.2.2. Performance Evaluation

In the performance evaluation, the ground truth of CRM is directly defined based on the phantoms for every simulated lesion size at each change-rate. The estimated CRM is compared to the corresponding ground truth for evaluating its quality. In this study, the indexes of image quality are adopted to objectively and systematically quantify the performance. Denote the value of the *i*th voxel in the estimated CRM as *R*_*i*_, and denote the value of corresponding voxel in ground truth as *G*_*i*_. Thus, the normalized absolute error (NAE), which is the simplest metric for measuring the difference between two images, can be calculated by

(4) $$\begin{array}{c} NAE=\frac{\sum_{i=1}^{N}\left|R_{i}-G_{i}\right|}{\sum_{i=1}^{N}G_{i}}. \end{array}$$

As shown in (5), the peak signal to noise ratio (PSNR) is the index to reflect the image quality of obtained CRM comparing with its ground truth.

(5) PSNR=20 log10GmaxMSE,MSE=1N∑i=1NRi−Gi2,

where *G*_max_ is the maximum voxel value of ground truth of CRM; MSE is for mean square error.

The normalized cross-correlation (NCC) is used to quantify the similarity between the obtained CRM and its ground truth. The NCC can be calculated by

(6) $$\begin{array}{c} NCC=\frac{1}{N-1}\overset{N}{\underset{i=1}{\sum }}\frac{\left(R_{i}-\overset{-}{R}\right)\left(G_{i}-\overset{-}{G}\right)}{\sigma_{c}\sigma_{G}}, \end{array}$$

where $\overset{-}{R}$ and $\overset{-}{G}$ represent the mean values of the obtained CRM and the corresponding ground truth, respectively; *σ*_*c*_ and *σ*_*G*_ denote their standard deviations.

After the recovered region is detected based on CRM, the Dice similarity coefficient (DSC), which is calculated as in (7), is used to measure the accuracy of the recovered regions detection comparing with the ground truth that is the predefined sphere in the phantom.

(7) $$\begin{array}{c} DSC=2\times \frac{TP}{TP+FN+TP+FP}, \end{array}$$

where TP is true positive, that is, the set of voxels common to the derived recovered region and ground truth; TN is true negative, that is, the set of voxels not labelled as the derived recovered region and ground truth; FN is false negative; and FP is false positive.

Moreover, the change-rates derived from the recovered regions are also used to evaluate the accuracy of recovered region detection. Because of the homogeneity of voxel values in the predefined digital phantom, only the mean change-rate of the recovered region is calculated and compared with the predefined real value by linear regressions.

### 2.3. Clinical Applications

#### 2.3.1. Clinical Data Acquisition

In this study, ^99m^Tc-ECD SPECT brain imaging is used in the treatment monitoring of the internal carotid artery (ICA) stenting, which is a common treatment technique for brain ischemia. This study has been approved by the Ethics Committee of Renji Hospital, School of Medicine, Shanghai Jiaotong University. All the SPECT scans are performed in accordance with the guidelines for brain perfusion SPECT using ^99m^Tc-labelled radiopharmaceuticals [1]. 50 patients in total (7 women, 43 men, and average age 62.9 ± 10.5 years) prescribed ICA stenting are recruited. 27 of them have cerebral infarction, while the rest suffer different degrees of cerebral ischemia. For each patient, the baseline ^99m^Tc-ECD SPECT imaging is performed within 7 days before surgery, and then the follow-up scan is generally conducted in ~7 days (ranged from 2 to 12 days) after the treatment of ICA stenting. The SPECT imaging was started within 20~30 minutes after the radiotracer injection (around 25 mCi ^99m^Tc-ECD) using dual-head Philips Precedence 6 SPECT/CT scanner with low-energy and high-resolution collimators. For 41 patients, the CT scans are performed together with SPECT imaging. For the rest of 9 patients, the corresponding CT images are not available. The system resolution is 7.4 mm full width half maximum (FWHM) at 10 cm for SPECT imaging. Three-dimensional SPECT images were reconstructed using Astonish™ technology, which adopts an iterative ordered-subset expectation-maximization (OSEM) algorithm with built-in scatter correction and attenuation correction [24]. For each patient, a pair of baseline and follow-up SPECT images was used to evaluate the therapy of ICA stenting.

#### 2.3.2. Clinical Data Analysis

For the data analysis, the traditional visual inspection and the CAA-CRM approach are both used to assess the recovery levels. For the visual inspections, the baseline and follow-up SPECT brain images were compared by two independent experienced physicians in the same image workstation. When CT images are available, the physicians inspected the SPECT images with the support of the aligned CT images. The hypoperfusion lesions caused by cerebral infarction or ischemia are carefully studied, and then the recovered regions are delineated manually. Furthermore, the overall recovery level for each patient is formally reported in four scales (none, mild recovery, moderate recovery, and severe recovery) based on the physicians' experience. On the other hand, these longitudinal SPECT images are quantitatively analyzed by the CAA-CRM approach. After all automatic processes, a CRM is derived for each patient. Then, the estimated CRM is fused with the original SPECT/CT images. The recovered regions are automatically derived based on the CRM by thresholding and clustering. The threshold is set as 20% according to the results of computer simulations (as mentioned in Section 3). Small regions (less than 120 voxels) are excluded to eliminate the influence of measurement noise. Then, the automatically recovered regions are located by the atlas of brain lobes and the quantitative indexes related to recovery level are estimated. In the further evaluation of the performance of CAA-CRM approach in treatment monitoring, the clinical diagnosis derived by visual inspection is considered as the standard of the classification of recovery groups to validate the results of the CAA-CRM approach.

In the performance evaluation, the automatically recovered regions are firstly compared with manually defined ones. If an automatic recovered region hits the corresponding manual one, it would be considered as a successful detection of the real recovered region. Moreover, McNemar's test [25] for paired automatic and manual recovered regions is used to investigate the concordance of the CAA-CRM approach and traditional visual inspection in the detection of recovered regions. Meanwhile, quantitative indexes, including the mean change-rate, maximum change-rate, and proportion of the recovered regions to the corresponding lobes, are estimated from the CRM. The statistics of these quantitative indexes related to the recovery levels are analyzed in three different recovery groups classified according to clinical reports. Nonparametric one-way ANOVA is also applied in the analysis of variance of different recovery groups.

## 3. Results

### 3.1. Results of Computer Simulation

**Figure 2:**
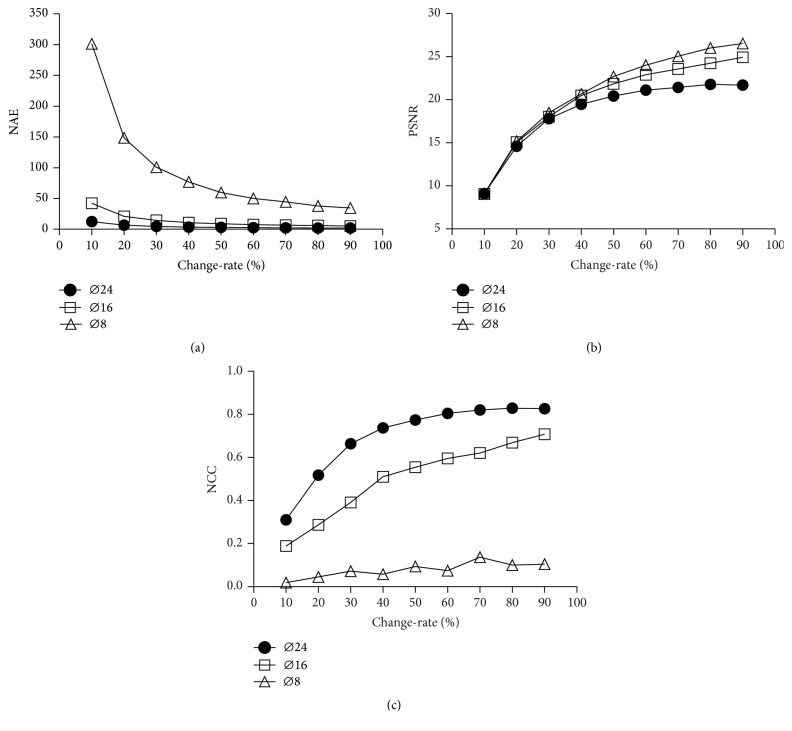
Comparisons of the image quality indexes for three-size lesions (*ϕ*8 mm: diameter of 8 mm, *ϕ*16 mm: diameter of 16 mm, and *ϕ*24 mm: diameter of 24 mm) at nine different change-rates (10% to 90%). (a) The normalized average error (NAE) for measuring the difference between the change-rate map (CRM) and its ground truth; (b) the peak signal-noise ratio (PSNR) for reflecting the image quality of the CRM; (c) the normalized cross-correlation (NCC) for quantifying the similarity between the CRM and its ground truth.

Using CAA-CRM approach, the CRMs are derived based on the simulated data. These estimated CRMs are compared with the corresponding ground truths. The properties of CRM in treatment monitoring are quantified by image quality indexes, including the NAE, PSNR, and NCC. The comparative results of the image quality indexes for different lesion sizes at each change-rate are shown in Figure 2. In Figure 2(a), NAE declines with the increase of change-rate for each lesion size. Moreover, for small-size lesion (*ϕ*8 mm), the calculated NAE is much higher than larger-size lesions (*ϕ*16 mm and *ϕ*24 mm), especially in the condition of low change-rates (10% and 20%). In Figure 2(b), PSNR progressively rises along the increase of change-rate, and it climbs quicker in the case of small-size lesion.  Figure 2(c) shows that NCC goes up with the increase of change-rate when the lesion size is comparatively large (*ϕ*16 mm and *ϕ*24 mm). However, NCC increases slightly for small-size lesion.

**Figure 3:**
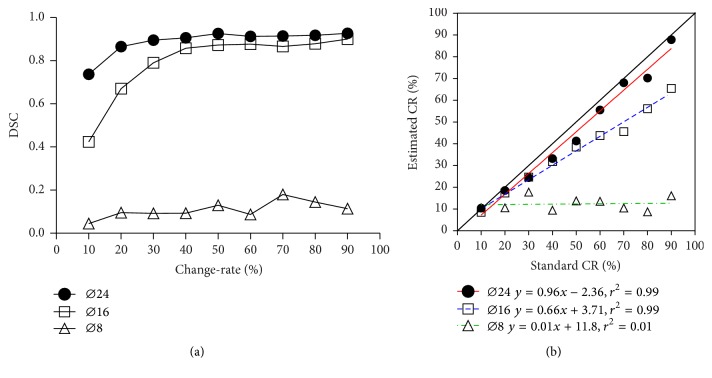
Comparisons of detected recovered regions with the ground truth for three-size lesions (*ϕ*8 mm: diameter of 8 mm, *ϕ*16 mm: diameter of 16 mm, and *ϕ*24 mm: diameter of 24 mm) at nine different change-rates (10% to 90%). (a) The Dice similarity coefficient (DSC) for measuring the accuracy of the recovered regions detection comparing with the ground truth; (b) linear regressions of estimated change-rates (CR) of the recovered regions with the real values.

DSC and mean change-rate estimates of recovered region are both used to evaluate the performance of the derived CRM in recovered region detection. From the curve chart in Figure 3(a), the DSC of large lesion (*ϕ*24 mm) maintains a higher value (>0.7). For the medium lesion (*ϕ*16 mm), the DSC increases quickly with rising of change-rate when the change-rate is less than 40%. Meanwhile, the DSC changes slightly and stays in a high level when the recovery level is higher than 40%. However, the DSC fluctuates with a low value along the increase of recovery level for the small lesion (*ϕ*8 mm). In Figure 3(b), the linear regressions are plotted for three different lesion sizes. The highly linear relations (*r*^2^ = 0.99, *p* < 0.0001) between the change-rates estimates and real values are observed in the condition of larger lesions (*ϕ*16 mm and *ϕ*24 mm). It is also found that the estimated mean change-rates are underestimated since the liner regression lines of the estimates and real values are under the line *y* = *x* (the black solid line). The underestimation is more serious for the small lesions. It seems that the CAA-CRM approach fails in deriving the acceptable change-rate for small-size lesion with diameter of 8 mm.

Based on the results of compuater simulations, it can be concluded that the CAA-CRM approach has a better performance in the detection of recovered regions and the quantification of change-rates, when the lesion size is sufficiently large (larger than a sphere with diameter of 8 mm that is closed to the proposed spatial resolution of SPECT images) and the change-rate is high enough (at least not lower than 20%).

### 3.2. Results of Clinical Application

For the visual inspection, 17 of 50 patients are reported as severe recovery in brain perfusion after ICA stenting, while 22 patients are scaled as moderate recovery. In the remaining 11 patients, there are 8 patients with mild recovery, and 3 patients are diagnosed as no improvement along with the treatment. Considering the population distributions of these 4 groups, the mild and none recovery patients are combined as one group to compare with the other groups. In the visual inspection, 106 manual VOIs are totally delineated to locate the recovered regions for these 50 patients.

**Figure 4:**
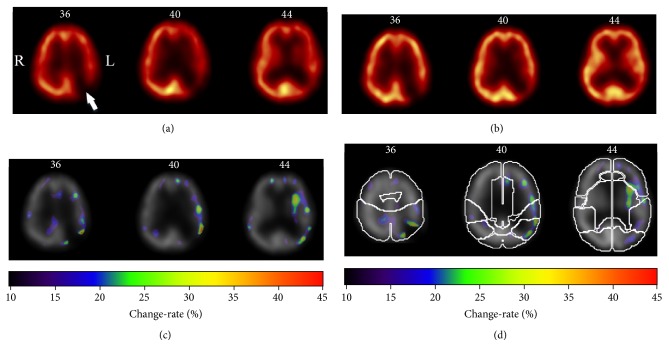
A typical case of treatment monitoring using CAA-CRM approach, where the estimated change-rate map can directly reflect response to treatment. The patient (male, 69-year-old) suffered cerebral infarction in left parietal lobe. While the baseline ^99m^Tc-ECD SPECT scan was performed 3 days before the treatment of ICA stenting, the follow-up scan was obtained 7 days after the treatment. (a) Three transverse slices (the 36th, 40th, and 44th slices) of baseline SPECT images. The lesion of cerebral infarction is pointed out by white arrow. Around this lesion, the hypoperfusion could be observed. (b) Three corresponding transvers slices of the follow-up SPECT image. There is no improvement in the lesion of cerebral infarction, while the severe recovery is observed for the cerebral ischemia in hypoperfusion regions around the lesion. The recovery level was scaled as severe by the traditional visual inspection in clinical report. (c) Three transverse slices of the derived change-rate map (CRM) fused with baseline SPECT image. The scales of change-rate are presented by rainbow color bar. The warmer color denotes higher change-rate. In this case, the recovered regions can be detected easily and clearly in the change-rate map. (d) The selected transverse slices of the CRM fused with the atlas of brain lobes. The contours of the brain lobes are delineated. The recovered regions can be conveniently localized in the brain area.

Beside the visual inspection, the CAA-CRM approach is applied to monitor the changes based on the longitudinal SPECT images for each patient.  Figure 4 illuminates a typical case of severe recovery. In Figure 4(a), there are three transverse slices (the 36th, 40th, and 44th slices) of baseline ^99m^Tc-ECD SPECT images. The lesion of cerebral infarction can be clearly found in the left parietal lobe, which is pointed by a white arrow. The cerebral ischemia is easily detected for the hypoperfusion regions around the lesion. The corresponding slices of aligned follow-up SPECT images are shown in Figure 4(b). In the clinical report based on visual inspection, the lesion of cerebral infarction had no improvement after the treatment of ICA stenting. However, the cerebral blood flow recovers significantly in the hypoperfusion regions around the lesion of cerebral infarction. The overall recovery level given by physicians is severe recovery. In Figure 4(c), the estimated CRM presents as a parametric image fused with the corresponding baseline SPECT image. In this case, the derived CRM could be used to enhance the visual inspection for physicians in treatment monitoring. In Figure 4(d), the CRM as well as the corresponding SPECT images is mapped to the standard atlas of brain lobes for the convenient localization of recovered regions.

The CAA-CRM approach also has the advantages in automatic and quantitative analysis of recovered regions. For all the patients' SPECT images, the recovered regions are automatically derived, located, and compared to manual ones which are used as the standard for validation. The detailed results are listed in Table 2 to reflect the concordance between the automatic detection and manual detection of recovered regions. From Table 2, there are in total 99 concordat pairs (automatically recovered regions that hit manual ones) and the total concordance rate of localization is 93.4%. For the rest of 19 discordant pairs, there are 12 (12/19, 63.2%) pairs where the automatic method recommended recovered regions while the physicians did not, and 7 (7/19, 36.8%) pairs are in the contrary condition. By the conventional criteria of McNemar's test (*p* < 0.05), this difference is considered to be not statistically significant. There is also no significant difference between the automatic and manual approaches in localization of recovered regions, whatever group (severe, moderate, and mild/none groups) is chosen. The results indicate that the CAA-CRM approach and visual inspections of experienced physicians have the concordance in the localization of recovered regions.

**Figure 5:**
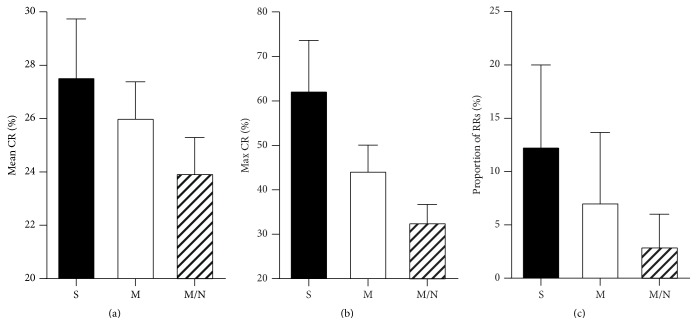
The bar graphs (mean + SD) for comparisons of quantitative indexes for three recovery groups (S: severe, M: moderate, and M/N: mild/none). (a) The mean change-rate (CR) estimates of the recovered regions; (b) the maximum CR estimates of the recovered regions; (c) the proportion of recovered regions (RRs) to the corresponding brain lobes.

After localizing the recovered regions, the mean and maximum change-rates for each patient could be calculated based on the delineated recovered regions and then compared in groups. The group-wise results of statistical comparisons are illuminated by bar graphs in Figure 5.  Figure 5(a) illuminates that the mean change-rates of the severe recovery group are mainly higher than those for the moderate group and mild/none group. The similar results can be observed in Figure 5(b) for the maximum change-rate estimates for three groups. The statistical results of nonparametric one-way ANOVA indicate that the significant differences (*p* < 0.0001) exist among the mean/maximum change-rates of three different recovery groups. Beside the indexes of change-rate, the proportion of recovered regions to the corresponding brain lobes, which is calculated as a volume ratio between recovered regions and the corresponding brain lobes, is also a specific index for quantifying the recovery level for treatment monitoring. The comparison of proportion of recovered regions for three recovery groups is shown in Figure 5(c). The tendency of the proportions in groups is similar to that of the change-rate. It is obvious that the better recovery groups have the higher proportions. According to the results of nonparametric one-way ANOVA, three recovery groups had significant effect (*p* < 0.001) on proportion of recovered regions to the brain lobes.

To sum up the results of clinical applications, the higher change-rates and larger recovered regions could correspond to better recovery levels given in the clinical reports. These quantitative indexes derived by the CAA-CRM approach can be used to quantify the response to the treatment.

## 4. Discussion

In this study, the performance of the CAA-CRM approach is objectively and systematically evaluated by the computer simulations as well as the clinical applications. From the results of computer simulations, the lesion size and change-rate are considered as two major factors for extracting reliable CRM. According to the changing tendency of image quality indexes of CRM, it can be concluded that the lesions with larger size and higher change-rate could be easier to detect; moreover, the estimates of change-rate could be more accurate as the real values. This conclusion confirmed our experiences from clinical practice. Additionally, the results also clarified that the spatial resolution of SPECT image could be a major limitation of the CAA-CRM approach to get accurate quantitative indexes for quantifying the recovery levels. It has been reported that the quantitative accuracy of the radioactive concentration in SPECT image would deteriorate with the decreasing of target size, especially when the targets are below three times of spatial resolution [26]. For the CAA-CRM approach, the poor quantitative accuracy of original SPECT images would directly result in estimated bias of change-rate and even in the missing recovered regions. As the results shown in Figure 2(c), for the small lesion (*ϕ*8 mm), whose size is closer to spatial resolution (7.4 mm FWHM at 10 cm), the values of NCC are much lower than those of the larger lesions (*ϕ*16 mm and *ϕ*24 mm). The uptrend with increasing change-rate is also very slight. The low value of NCC reflects the poor similarity between the obtained CRM and its ground truth. It means that the obtained CRM could not accurately reflect the small recovered regions when the region size is closed to or even smaller than the spatial resolution of SPECT images.

In the computer simulations, the underestimation of change-rate is observed for all lesions with varied sizes. However, the underestimation is more significant for the small lesion. The bias probably comes from the partial volume effects (PVEs) that are usually related to the spatial resolution. The PVEs could induce the underestimation for quantitative SPECT images, especially when the target is smaller than three times of spatial resolution [26]. This impact could directly propagate into the CRM that is derived based on SPECT images. As illuminated in Figure 3(b), the estimated change-rate is much lower than the predefined change-rate for the small-size lesion (*ϕ*8 mm). The estimated change-rates for middle-size lesion (*ϕ*16 mm) have the high linear relation with the predefined values, although the values are only nearly 70% of the real values. It is concluded that the change-rate could be significantly underestimated comparing with the real value, when the sizes of recovered regions are below three times of spatial resolution. Therefore, this underestimation should be kept in mind for clinical applications.

For the clinical applications, the CAA-CRM approach could be used to define the recovered regions and derived quantitative indexes to measure recovery levels. In this study, the thresholding and clustering method is applied to automatically derive the recovered regions. The chosen threshold of change-rate could be the major impact factor in delineating the recovered regions. Furthermore, it could influence the estimations of quantitative indexes from recovered regions [27, 28]. The experimental threshold is set as 20% in the clinical applications. It is chosen based on the results of the computer simulations. As shown in Figure 2, the image quality indexes for the change-rates of 10% and 20% are both much poorer than the other change-rates regardless of the lesion size. The CRM would not accurately reflect the change-rate lower than 20%. This indicates that the significant distortions may exist in the CRM for the voxels with lower change-rates. In this case, the lowest limit for available estimated range of change-rate is required to eliminate the turbulences from lower change-rate estimates. Meanwhile, the threshold setting should try to retain as much information as possible in the CRM for further quantitative analysis. Hence, the experimental threshold set in this study is chosen as 20% rather than 10%. The thresholds could also be able to reset for different conditions of varied clinical applications.

**Figure 6:**
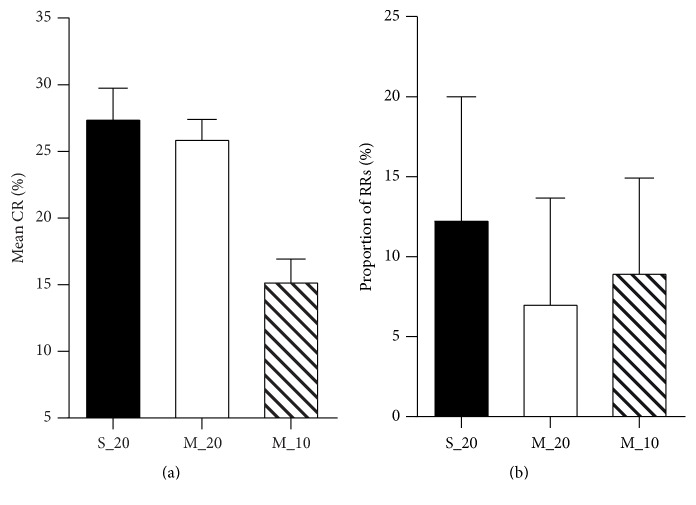
The bar graphs (mean + SD) for comparisons of quantitative indexes for severe and moderate recovery groups with different thresholds (S_20: severe recovery group with the threshold of 20%, M_20: moderate recovery group with the threshold of 20%, and M_10: moderate recovery group with the threshold of 10%). (a) The mean change-rate (CR) estimates of the recovered regions; (b) the proportion of recovered regions (RRs) to the corresponding lobes.

In the clinical applications, considering the concordance of the CAA-CRM approach in the detection of recovery regions, the false positive (12/99) and false negative (7/99) could be found. The false positive might be caused by using the uniform 20% threshold which might result in the detection of not only the main recovered regions (validated by visual inspections) but also the other minor recovered regions. However, the minor recovered regions might be ignored by the physicians. On the other hand, the uniform threshold could easily introduce the small changed regions (<120 voxels) for a certain case. The small changed regions could be removed or even miss the main recovered regions. This may lead to the false negative. In this condition, the threshold should be adjusted carefully to balance the false positive and false negative for detecting the recovered regions. Moreover, the chosen threshold could further impact on the estimations of the mean change-rate and the proportion of recovered regions to the brain lobes. As shown in Figure 6, when a threshold of 10% instead of 20% is applied in the moderate group, the values of mean change-rate are decreased sharply. Meanwhile, the values of proportion of recovered regions to the brain lobes have increased. From the comparisons, the mean change-rate is negatively related to the proportion of recovered region. Therefore, these two quantitative indexes should be used together to scale the recovery level in treatment monitoring. To an extent the maximum change-rate could not be affected by the chosen threshold in the CAA-CRM approach, so that it becomes an important quantitative index in treatment monitoring.

Because all the algorithms used in the proposed CAA-CRM approach totally rely on image contents, this approach could be extended to analyze other types of SPECT brain images, such as ^99m^Tc-HMPAO SPECT brain images. The obtained change-rate map could also illuminate the global changes for reflecting the response to treatment. The derived quantitative indexes have the potential to quantify the recovery levels.

## 5. Conclusion

In this study, a CAA-CRM approach has been introduced to evaluate the longitudinal ^99m^Tc-ECD SPECT images in treatment monitoring. This approach can provide change-rate map as a parametric image to reflect the changes of rCBF. Computer simulations show the efficacy of the proposed approach in detecting the recovered regions and in quantifying the change-rates for the lesions larger than spatial resolution. In clinical applications, this method is used to assess the treatment of ICA stenting. The results demonstrate that the quantitative indexes derived from CRM are all significantly different among the groups and highly correlated with the experienced clinical diagnosis.

In conclusion, the CAA-CRM approach has the advantages of directly illuminating the global recovery and conveniently quantifying the recovery levels. It could be helpful in improving the efficiency and accuracy of therapy evaluations using SPECT brain images in clinical routines.

## Figures and Tables

**Table 1:** The list of brain anatomical structures in the atlas of brain lobes.

Brain anatomical structures	Values in atlas
Cerebellum anterior lobe	80
Cerebellum posterior lobe	10
Frontal lobe	Left: 50, right: 55
Frontal-temporal space	Left: 110, right: 115
Limbic lobe	Left: 40, right: 45
Medulla	20
Midbrain	100
Occipital lobe	Left: 90, right: 95
Parietal lobe	Left: 120, right: 125
Pons	70
Sublobar	60
Temporal lobe	Left: 30, right: 35

**Table 2:** The concordance of automatic VOIs with manual ones in the localization of recovered regions.

	FN	TP	FP
Sever	3	41	5
Moderate	3	42	3
Mild/none	1	16	4
Total	7	99	12

FN: false negative, the number of manually recovered regions which were not hit by automatic ones; TP: true positive, the number of manually recovered regions which were hit by automatic ones; FP: false positive, the number of automatically recovered regions which could not find the paired manual ones.
